# Cerium as corrosion inhibitor for Sn–3Ag–0.5Cu solder alloy in 3.5% NaCl solution

**DOI:** 10.1038/s41598-026-44525-1

**Published:** 2026-03-18

**Authors:** R. Vani, Girish Kumar, Sathyashankara Sharma, Ananda Hegde

**Affiliations:** 1 Department of Mechanical Engineering, Yenepoya Institute of Technology (Affiliated to Visvesvaraya Technological University, Belagavi), Moodbidri, Karnataka 574227 India; 2 Department of Mechanical Engineering, SDM Institute of Technology (Affiliated to Visvesvaraya Technological University, Belagavi), Ujire, Karnataka 574240 India; 3 Manipal Institute of Technology, Manipal Academy of Higher Education, Manipal, 576104 Karnataka India

**Keywords:** Corrosion, Cerium, Solder, Chemistry, Materials science

## Abstract

The investigation was conducted to study the corrosion inhibition performance of cerium as an inhibitor for Sn–3Ag–0.5Cu (SAC305) lead-free solder alloy in 3.5% NaCl solution. The investigation is useful to suggest an inhibitor for SAC305 solder for microelectronic applications in marine environments. Weight loss measurement results for 30 °C showed the maximum inhibition efficiency as 33.33% when 700 ppm of cerium were added to 3.5% NaCl solution. However, polarization experiments displayed a better corrosion resistance for the alloy in 3.5% NaCl solution at 700 ppm of inhibitor concentration almost at all higher temperatures (40 and 50 °C). At room temperature, the corrosion rate decreases at 700 ppm, indicating that a high inhibitor concentration increases protective efficacy. Hence, cerium appeared to be a capable inhibitor for the alloy under the NaCl acidic environment.

## Introduction

A common metallurgical joining procedure involves soldering, which joins metallic components in combination using a filler metal. Lead-based solders have been commonly employed because of their low melting point, higher wettability, and low cost. However, lead’s toxicity and environmental risks have led to stringent global regulations and the development of lead-free substitutes^1^. Because of their suitable melting temperature, mechanical dependability, and compatibility with contemporary manufacturing processes, tin-silver-copper (SAC) alloys have grown into the most successful lead-free solders. SAC305 (Sn–3.0Ag–0.5Cu) has emerged as the most suitable material for electronic packaging applications^2^. Furthermore, studies indicate that SAC alloys with a copper content between 0.5% and 0.7% by weight are most effective when utilized instead of traditional Sn–Pb solders^3^.

Despite SAC alloys having good mechanical and thermal properties, their long-term durability in difficult environments—like high temperatures, humidity, and marine settings—remains a significant concern.

The oxidation and corrosion of solder joints can decrease their mechanical strength, raise electrical resistance, and eventually cause electronic components to fail^4^. Air pollution, moisture absorption from underfills materials, and the presence of chloride ions in maritime environments all exacerbate corrosion processes^3–5^. Under normal air circumstances, corrosion might be negligible, but in high-humidity or saline environments, like those encountered in coastal or aircraft applications, it becomes a major reliability risk^6^. With the goal to improve the environmental durability of lead-free solders, several studies have investigated their corrosion behavior. Sn–Ag alloys have shown good corrosion resistance in NaCl solutions, while adding Cu to SAC alloys, improve corrosion performance by improving the morphology and distribution of intermetallic compounds (IMCs)^5,7^. In this regard, Jaiswal et al. have made important contributions through a systematic series of electrochemical studies on Sn-based lead-free solders. According to their 2021 study on Sn–0.7Cu–xIn alloys, the incorporation of indium increased passive-layer stability and corrosion resistance by refining the microstructure and dramatically lowering corrosion current density (Icorr) in 3.5 weight% NaCl^8^. By strengthening the IMC network and encouraging the production of adherent oxide films, aluminum additions significantly improved corrosion resistance in subsequent research on Sn–In–Al ternary alloys^9^. In a similar vein, Sn–9Zn–xCu alloys have shown that higher Cu concentration improves electrochemical stability in saline conditions by altering the microstructure and reducing galvanic interaction between phases^10^. Corrosion resistance and solder junction reliability are improved by microstructural refinement through alloying or heat treatment, according to complementary studies on mechanical and environmental performance conducted by relevant research groups^11^. Collectively, the conclusions confirm that microstructural control through selective minor alloying is an effective way to improve the corrosion resistance of lead-free solders.

SAC305 solder’s vulnerability to corrosion has also been highlighted through electrochemical studies in alkaline and chloride-rich environments, emphasizing the need for advanced corrosion-prevention approaches^8^. One such approach involves the use of inhibitors that form protective surface films. Among these, cerium-based salts have attracted the attention for their ability to conquer corrosion while maintaining environmental safety^12^. Rare-earth (RE) elements such as neodymium (Nd) and cerium (Ce) have been shown to refine microstructure, inhibit galvanic coupling between IMCs (e.g., Ag₃Sn and Cu₆Sn₅), and lower corrosion current density^13^. Similarly, the addition of phosphorus (P) to Sn–Zn–Bi solders improve oxidation and corrosion resistance by soothing surface coatings^14^. Additional studies have established that Cu and Al additions to Sn–Zn and Sn–In base systems further enhance corrosion performance in chloride environments^9–11^.

However, despite these developments, studies specifically addressing Ce additions in SAC alloys remain inadequate. Prevailing reports propose that small Ce additions can refine the microstructure, hinder IMC growth, and form stable, adherent corrosion-resistant films^15–17^. However, the corrosion inhibition mechanism of Ce in SAC systems particularly in chloride-rich environments remains poorly understood.

To address this knowledge gap, the present study investigates the effect of cerium-based rare-earth inhibitors on the corrosion behavior of SAC305 solder in a 3.5 wt% NaCl environment, simulating high-humidity and marine exposure. Unlike prior studies that primarily focused on altering alloy composition, this work employs a sustainable chemical inhibition approach to enhance corrosion resistance without changing the base alloy. The key scientific contribution lies in representing that Ce ions facilitate the formation of protective, adherent surface layers that reduce corrosion current density and promote passivation. This approach represents a significant step toward developing more durable and environmentally responsible electronic assemblies by extending the lifetime and reliability of SAC305 solder joints in harsh environments.

## Materials and methods

### Materials

**Fig. 1:**
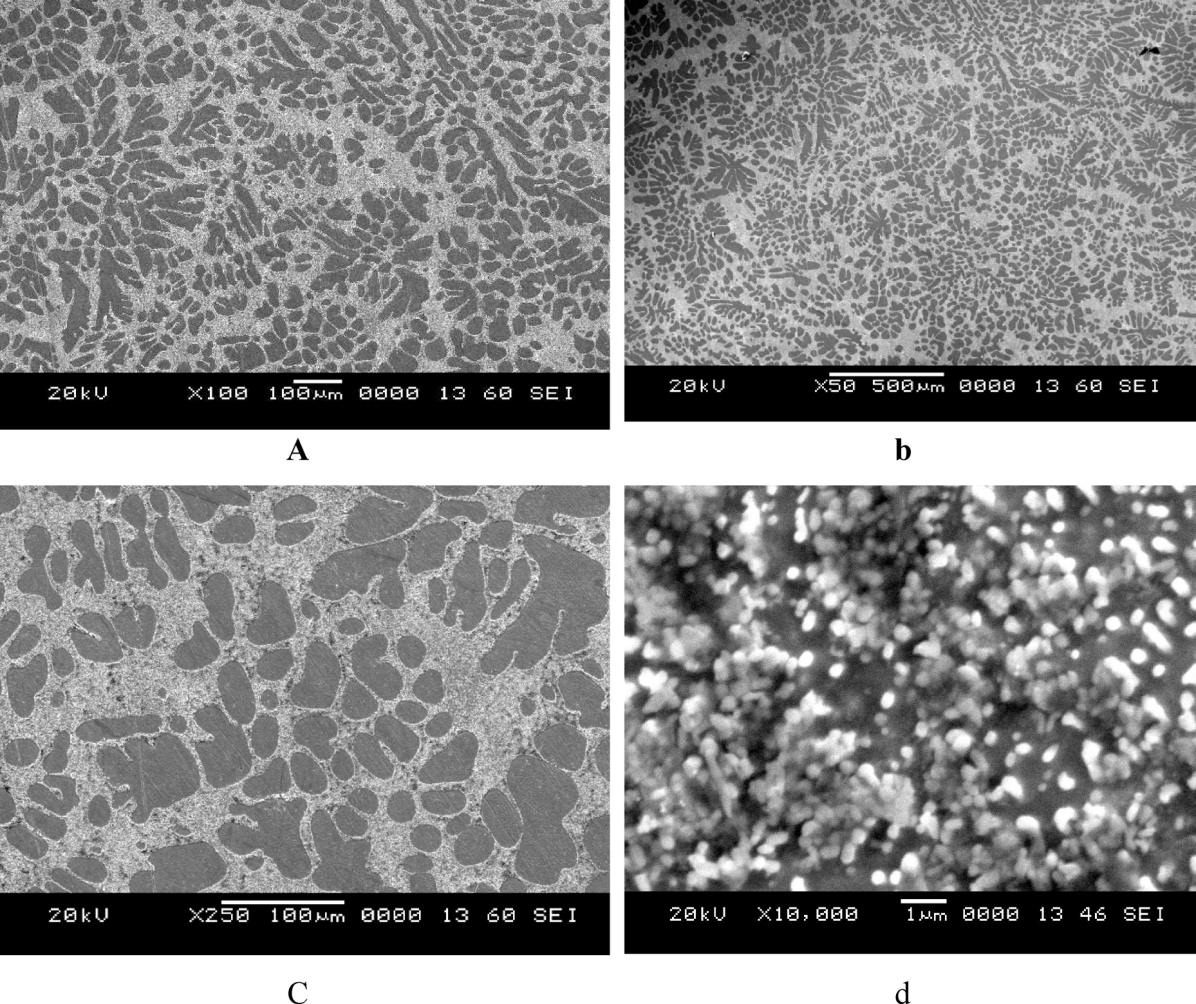
SEM images of SAC305 before immersion.

**Fig. 2:**
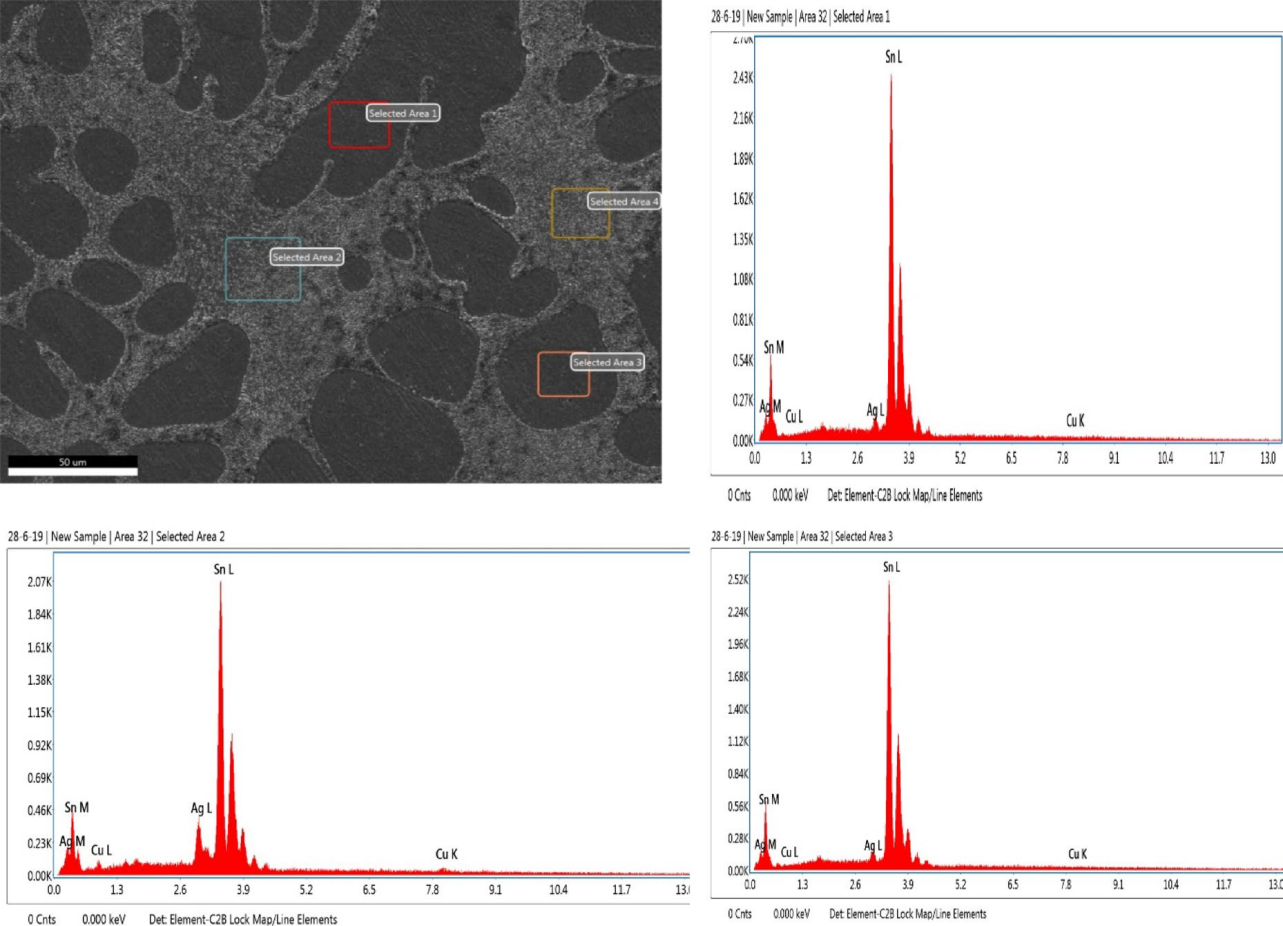
EDX Spectrum of SAC305 before immersion.

In this work, lead-free solder alloy SAC305 was used for investigation. The scanning electron microscope (SEM) images and EDX analyses of the sample before experimentation are presented in Figs. 1 and 2 respectively.

A β-Sn matrix with eutectic areas enhanced by intermetallic compounds (IMCs), mainly Ag₃Sn and Cu₆Sn₅, is evident in the SEM micrographs of SAC305. Higher amplifications reveal scallop-like Cu₆Sn₅ and acceptable Ag₃Sn dispersions, though lower magnifications show the dendritic β-Sn and interdendritic segregation of IMCs. These IMCs disturb the solder’s general electrochemical performance by serving as favourite positions for galvanic corrosion. The literature has predictable SAC305’s comparable microstructural characteristics^18^.

**Fig. 3:**
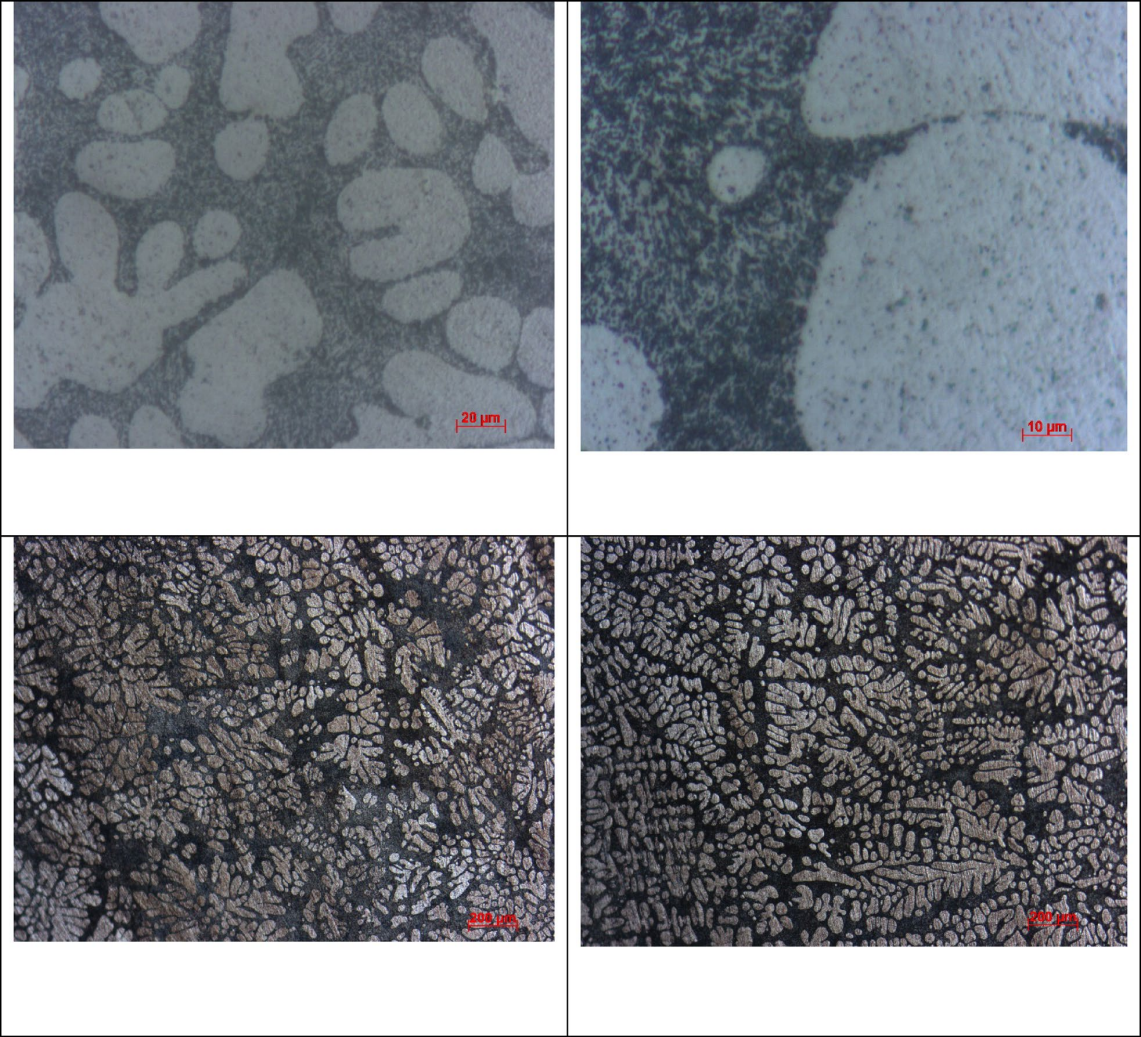
Microstructure of SAC305 before immersion.

The SAC305 alloy’s SEM-EDS study demonstrates a heterogeneous microstructure made up of intermetallic composites and a matrix rich in Sn. Through clear Ag and Cu peaks that show the construction of Ag₃Sn and Cu₆Sn₅ phases in the interdendritic zones, the EDS spectra from a few selected regions validate that Sn is the leading element. The distribution of these IMCs along the β-Sn dendrites is in line with the normal solidification behaviour of SAC305 as showed in Fig. 3. The compositional outcomes confirm that the distribution and arrangement of these intermetallic phases, which can aid as galvanic sites, are strictly linked to the alloy’s corrosion susceptibility^18^.

### Sample preparation

The spherical periphery rod like sample of SAC305 was mounted in cold mold by means of epoxy resin. The surface area of the specimen was measured to be 1.327cm^2^ area.

A hole was drilled on the sample, and it is joined with an operational electrode holder. Then the sample was cleaned by using Silicon Carbide sanding papers of different grades and then fine polished with velvet cloth with Al_2_O_3_ particles. The corrosive media with 3.5 wt% NaCl of 0.599 M solution was prepared by dissolving 3.5 g of NaCl in distilled water to make 100 mL, which approximately yield a neutral solution having pH 7.

### Inhibitor

Cerium was used as an inhibitor and is soluble in NaCl medium^19^. It was purchased from Shri Durga labs, Mangalore. And the specific gravity of cerium is observed to be 6.78. Cerium, the inhibitor was used in the range of 200–900 ppm for the weight loss experiments^20^. The solutions were vigorously shacked to ensure the uniform dispersion and full dissolution of the powder in the NaCl medium.

### Weight loss measurements

Weight loss experiments were performed by submerging the SAC305 solder alloy in a glass beakers containing 100 cm^3^ of 3.5% NaCl media with varied inhibitor proportions. As per the literature, 4 h of continuous dipping of the sample in the medium, the specimens were removed from the beaker and then cleaned with tap water then by pure distilled water, then completely dried and weighed accurately with digital balance to an accuracy: ± 0.1 mg. Each trial was conducted in static and aerated conditions. Trial was repeated thrice, and the average of readings was recorded. The weight loss technique was performed to regulate the suitable quantity of inhibitors important for conducting experiments for which the inhibitor works.

### Electrochemical measurements

Electrochemical corrosion of SAC305 solder alloy was explored in 3.5% NaCl medium. Gill AC potentiostat was used to measure the potentiodynamic polarization and impedance. A single cell by means of three normal electrodes of fully saturated Calomel Electrode as a reference electrode, platinum as a counter electrode and the sample as working electrode. In every case, the sample was allowed for 30 min in the trial solution to reach steady state open circuit potential (OCP). The Tafel curve of potentiodynamic current and potential were plotted by polarizing the sample (–250 mV cathodically and + 250 mV anodically with reference to the OCP at a scan rate of 1 mV/s). Corrosion potential (Ecorr), corrosion current (Icorr), anodic Tafel slope (*βa)* and cathodic Tafel slope (*βc*) were found with the software associated with the instrument. The Tafel plots are typically pitched in electrochemistry for several applications such as finding corrosion rate, assessing inhibitor efficiency etc.

Impedance readings were also found using AC signals of amplitude 5 mV at OCP in the frequency interval of 100 to 10 MHz. Nyquist plots were also plotted for the impedance parameters. Nyquist plots are used to analyse the corrosion tendency.

Electrochemical measurements are carried out at various temperatures since temperature is one of the significant features influencing both the rate of corrosion and mechanism of corrosion.

## Results and discussion

### Weight loss method

**Fig. 4:**
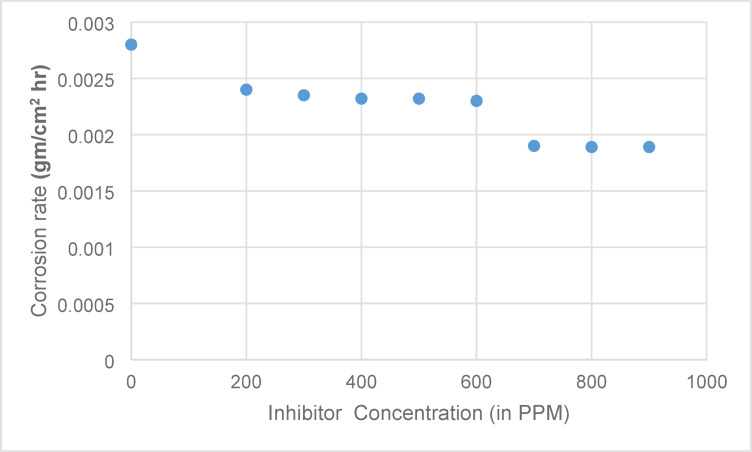
Effect of inhibitor concentration on corrosion rate.

The corrosion inhibition efficiency (*η*_*w*_) at various temperatures were studied with the help of weight loss technique^9^. *η*_*w*_ was calculated from the following relation.

$$\eta _{w} = \frac{{W_{o} - W}}{{W_{o} }}*100$$

where, *W*_°_ is the solder’s initial weight and W is the solder’s weight in the presence of inhibitor. The total amount of corrosion ρ (g cm^− 2^ h^− 1^) was found out by using the equation,

$$\rho = \frac{{W_{o} - W}}{{ST}}*100$$

where S denotes the exposed surface area of solder alloy, whereas T indicates the time of immersion. Table 1 summaries the parameters determined for various concentrations of cerium inhibitor. The effect of inhibitor concentration on the corrosion rate is shown in Fig. 4.

**Table 1:** Corrosion characteristics of SAC305 alloy in 3.5% NaCl solution, determined via weight loss method, at varying concentrations of cerium.

Corrosive Medium	Corrosion rate ρ (gm/cm^2^ hr)	η_w_
3.5% NaCl	0.0028	--
3.5% NaCl + 200 PPM of Cerium	0.0024	16.67
3.5% NaCl + 300 PPM of Cerium	0.00235	16.07
3.5% NaCl + 400 PPM of Cerium	0.00232	17.10
3.5% NaCl + 500 PPM of Cerium	0.00232	17.10
3.5% NaCl + 600 PPM of Cerium	0.00230	17.85
3.5% NaCl + 700 PPM of Cerium	0.0019	33.33
3.5% NaCl + 800 PPM of Cerium	0.00189	32.50
3.5% NaCl + 900 PPM of Cerium	0.00189	32.50

From the Fig. 4, it is observed that the weight loss decreases as the addition of cerium inhibitor in the 3.5% wt. of NaCl increases. Also, the *η*_*w*_ increases with the addition of Cerium quantity. The decline in corrosion rate with the increase in Cerium quantity is due to the adsorption of inhibitor molecules on the surface of the alloy. High *η*_*w*_ was reached at 700 ppm of the cerium inhibitor and beyond this quantity no changes were observed in *η*_*w*_. Hence, 700 ppm can be treated as the optimum quantity required to attain maximum inhibitor efficiency.

### Electrochemical measurements

#### Polarization measurements

**Fig. 5:**
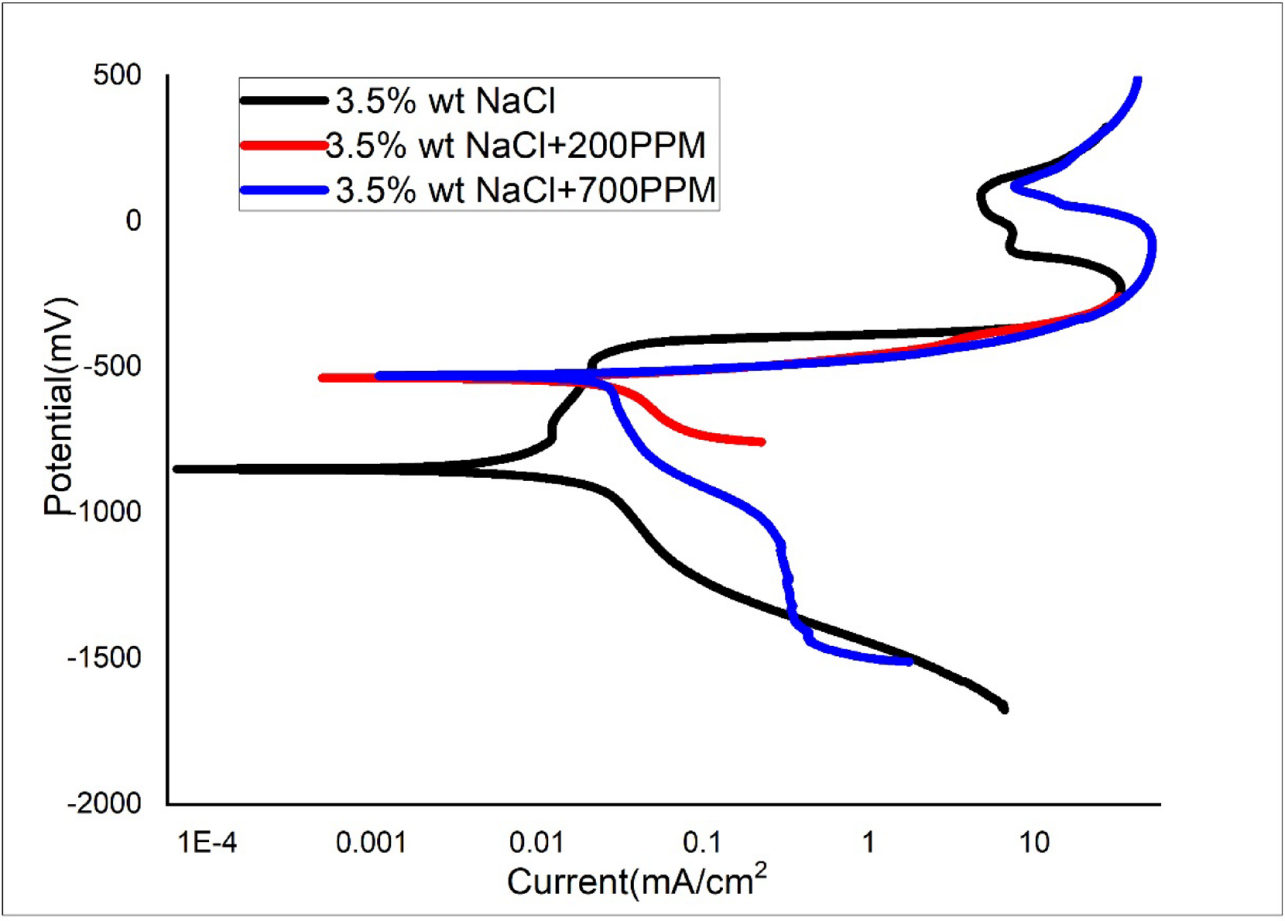
Tafel plot of SAC305 alloy in 3.5% NaCl at different concentrations of Cerium at 30 °C.

**Fig. 6:**
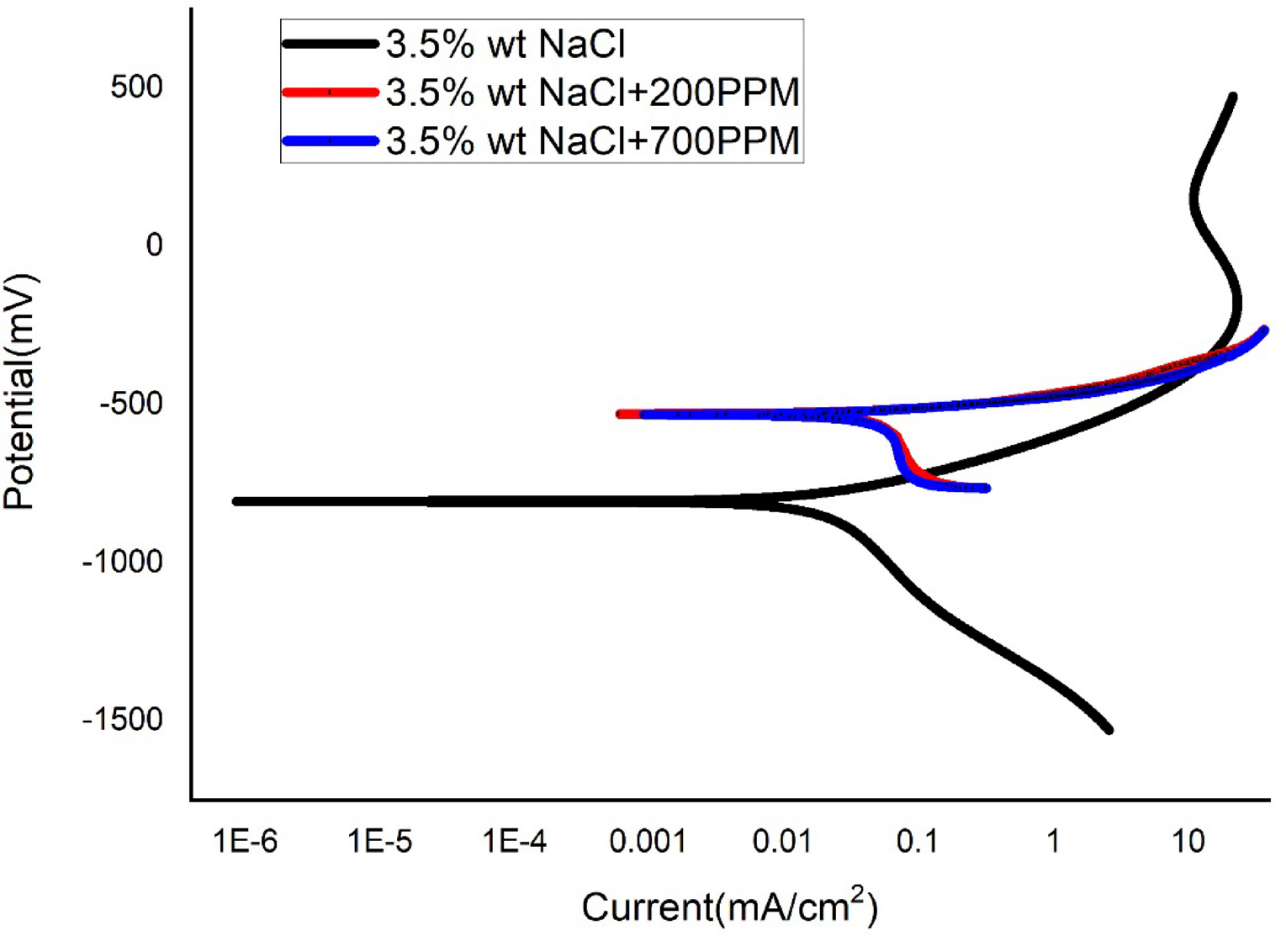
Tafel plot of SAC305 alloy in 3.5% NaCl at different concentrations of Cerium at 40 °C.

**Fig. 7:**
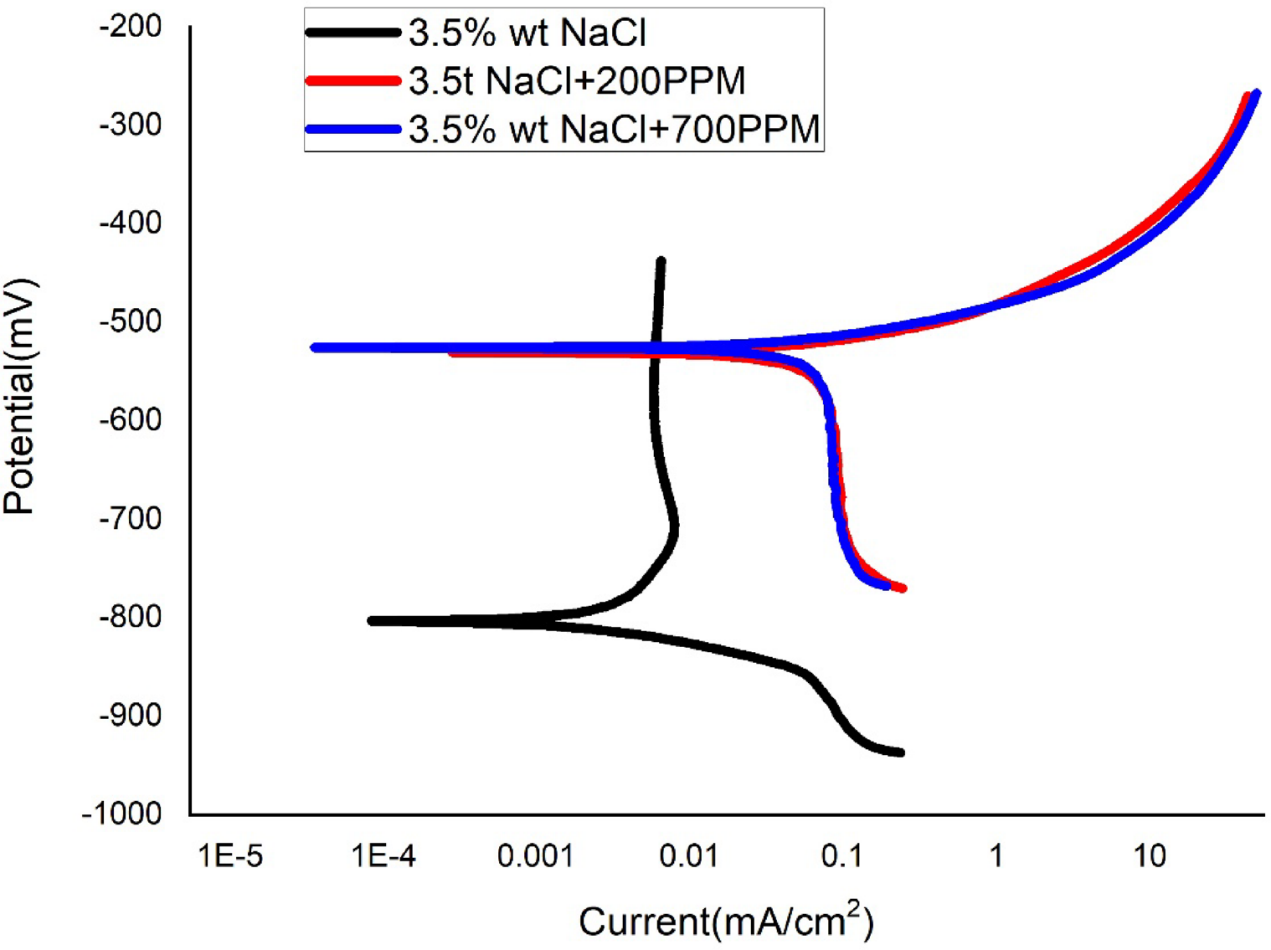
Tafel plot of SAC305 alloy in 3.5% NaCl at different concentrations of Cerium at 50 °C.

Figures 5, 6 and 7 are the cathodic polarization plots of SAC305 solder in NaCl medium with or without the presence of various amount of cerium at different temperatures. Corrosion current densities were obtained by the extrapolation of the curve’s linear parts to the corresponding corrosion potential. Tables 2, 3 and 4 summarize the polarization parameters including Ecorr, Icorr, βa, βc, corrosion rate, and inhibition efficiency obtained from Tafel extrapolation. η_w_ can be determined as,

$$\eta _{p} = \frac{{{\mathrm{I}}_{{{\mathrm{corr}}}}^{{\mathrm{o}}} - {\mathrm{I}}_{{{\mathrm{corr}}}} }}{{{\mathrm{I}}_{{{\mathrm{corr}}}}^{{\mathrm{o}}} }}*100$$

where I^o^_corr_, I_corr_ represents the corrosion currents in the absence and in the presence of cerium respectively^21^.

**Table 2:** Polarization data of SAC305 solder in 3.5% NaCl at different concentrations of Cerium at 30 °C.

Inhibitor conc. (ppm)	Rest Potential (mV)	I_corr_ (mAcm^− 2^)	Corrosion rate (m/y)	βc (mV)	βa (mV)	η_*p*_ (%)
0	−844.09	0.04	20.830	80.315	139.800	--
200	−550.96	0.007	3.65	120.027	140.222	82.5
700	−620.26	0.004	2.08	200.620	180.290	90.0

**Table 3:** Polarization data of SAC305 solder in 3.5% NaCl at different concentrations of Cerium at 40 °C.

Inhibitor conc. (ppm)	Rest Potential (mV)	I_corr_ (mAcm^− 2^)	Corrosion rate (m/y)	βc (mV)	βa (mV)	η_*p*_ (%)
0	−536.37	0.026	22.83	130.73	50.598	--
200	−529.99	0.012	10.43	80.033	40.938	53.8
700	−531.27	0.007	9.471	140.37	60.687	73.1

**Table 4:** Polarization data of SAC305 solder in 3.5% NaCl at different concentrations of Cerium at 50 °C.

Inhibitor conc. (ppm)	Rest Potential (mV)	I_corr_ (mAcm^− 2^)	Corrosion rate (m/y)	βc (mV)	βa (mV)	η_*p*_ (%)
0	−531.68	0.018	20.08	60.36	72.087	--
200	−534.09	0.011	12.3	72.52	40.295	40
700	−526.09	0.007	7.8	82.18	50.613	60

### Electrochemical behaviour and corrosion inhibition performance

**Fig. 8:**
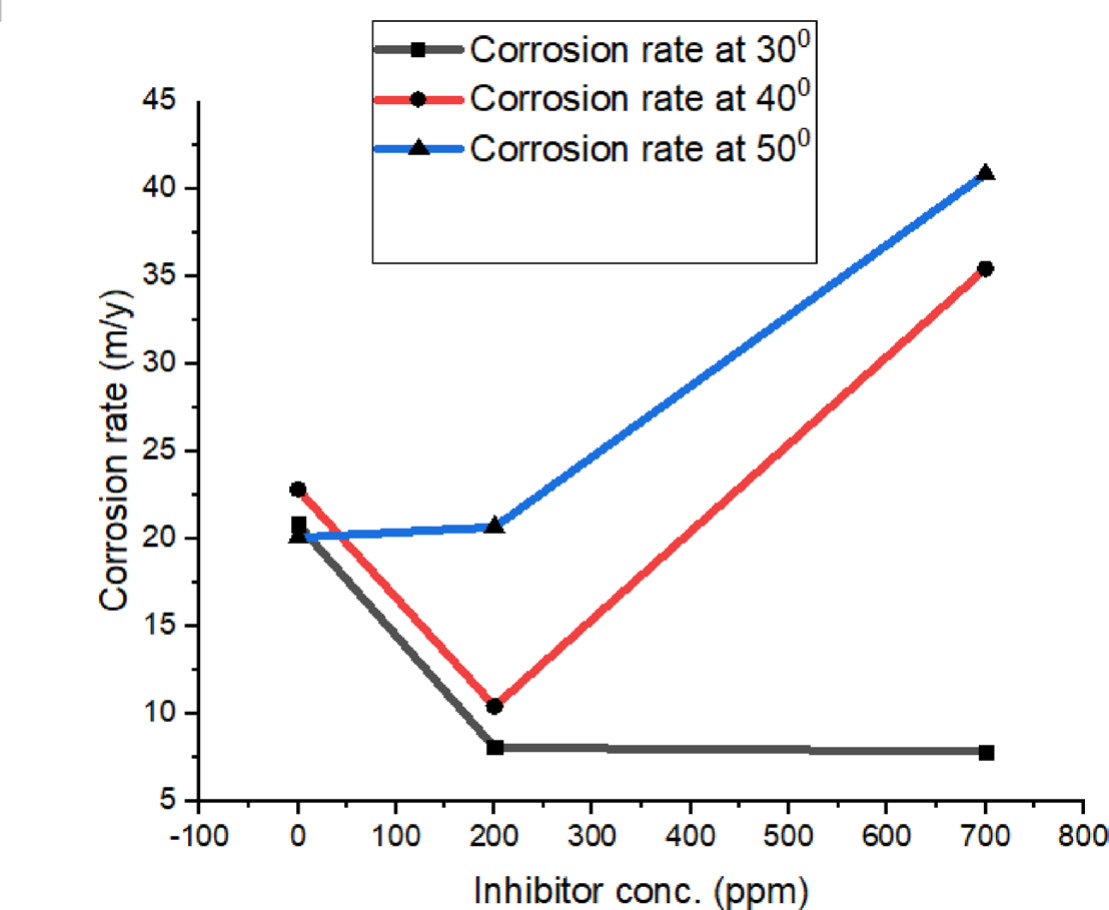
Effect of inhibitor concentration on corrosion rate at different temparatures.

**Fig. 9:**
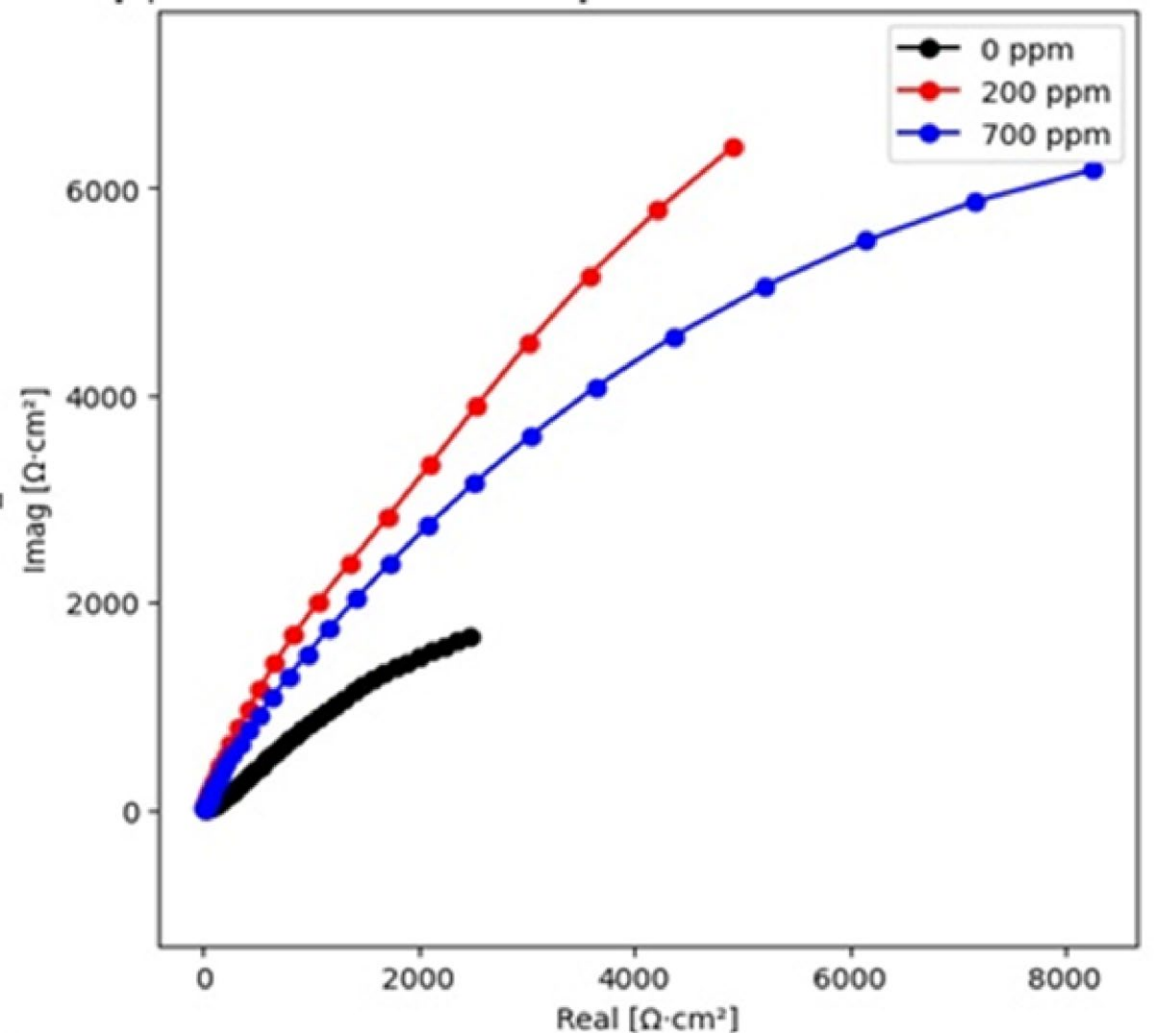
Nyquist Plot for SAC305 alloy in 3.5% NaCl at different concentrations of Cerium at 30 °C.

**Fig. 10:**
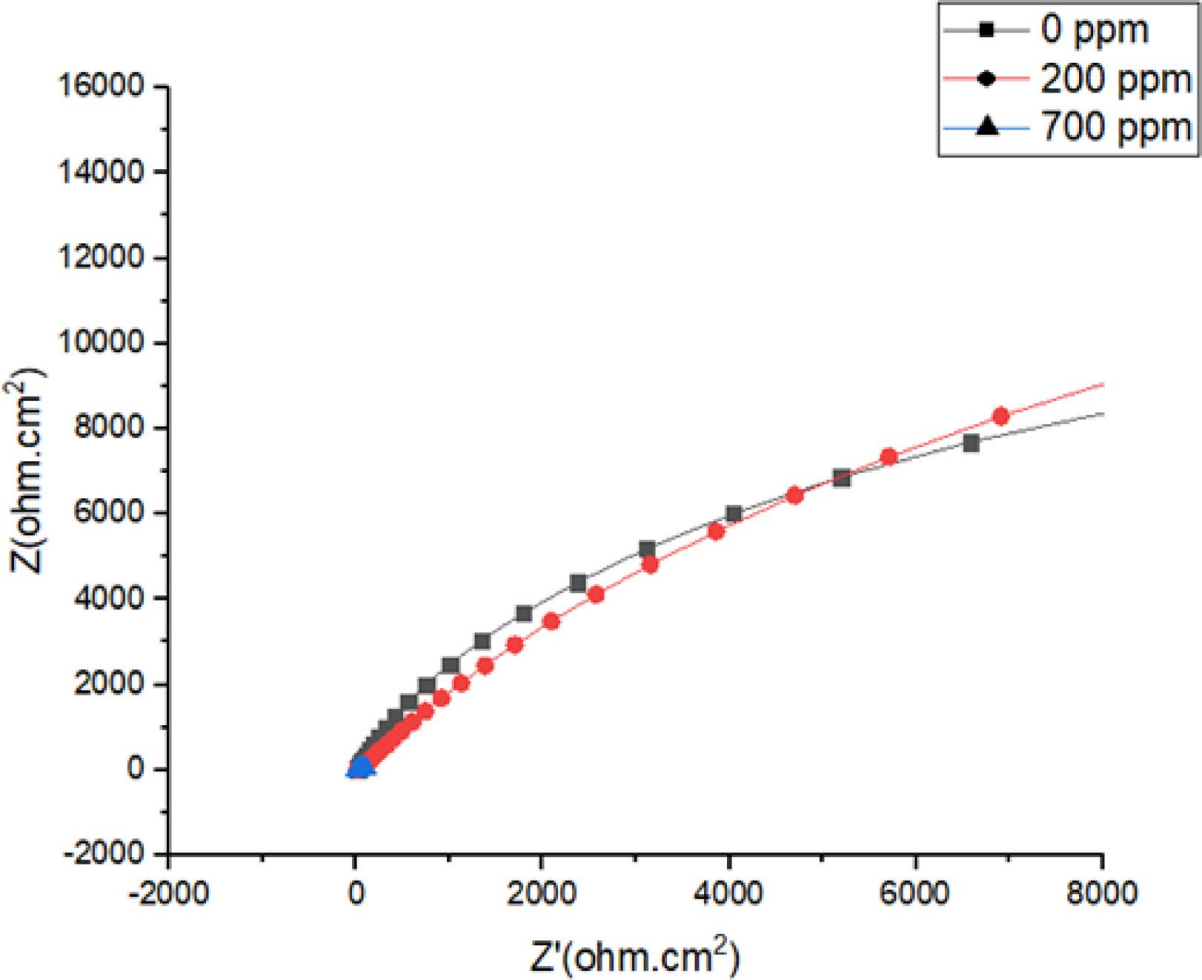
Nyquist Plot for SAC305 alloy in 3.5% NaCl at different concentrations of Cerium at 40 °C.

**Fig. 11:**
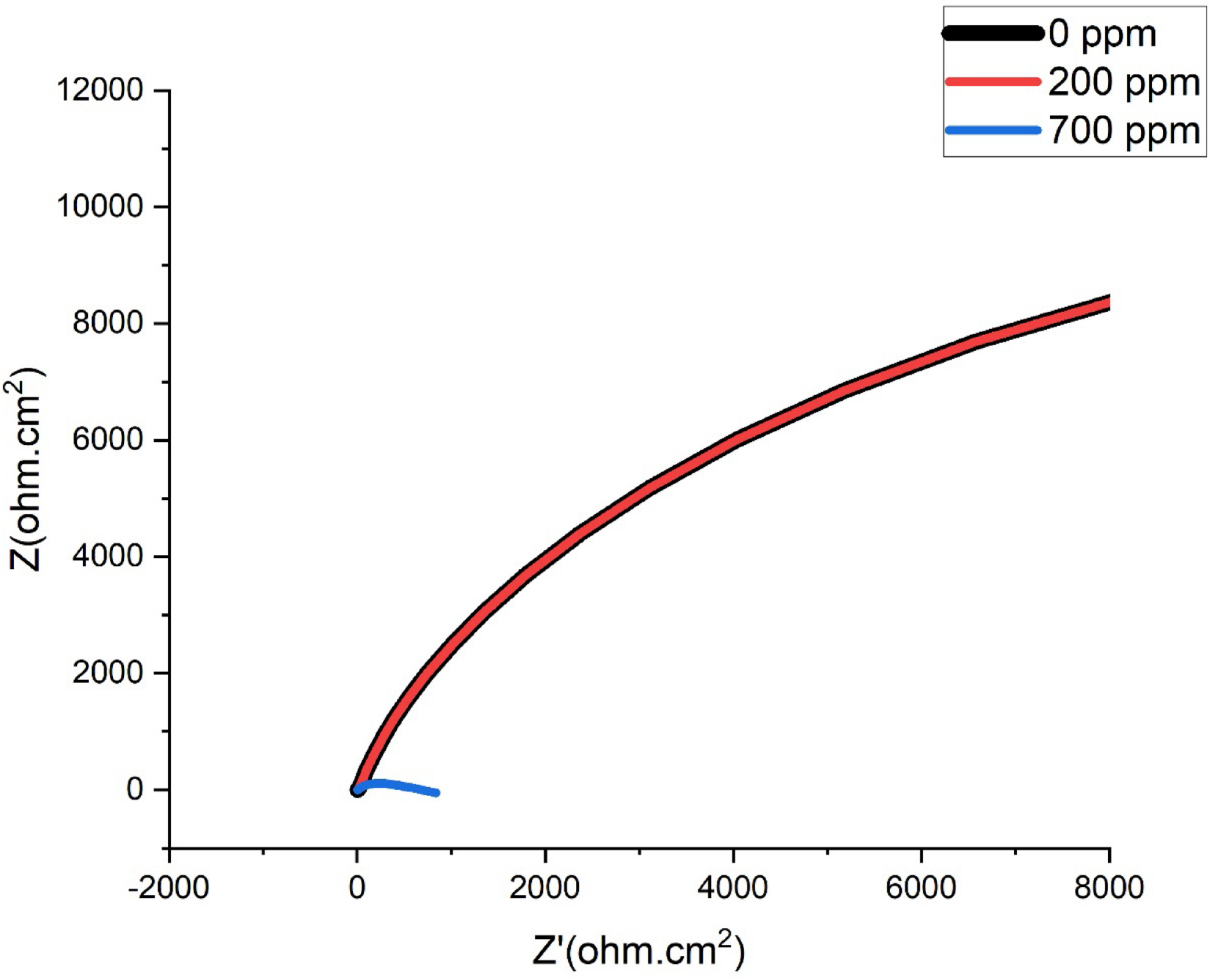
Nyquist Plot for SAC305 alloy in 3.5% NaCl at different concentrations of Cerium at 50 °C.

At elevated temperatures, the electrochemical parameters generally display behavior consistent with effective inhibition, as evidenced by positive inhibition efficiencies and reduced corrosion current densities in the presence of cerium-based inhibitor. However, the extent of improvement varies with temperature and inhibitor concentration. These observations suggest that, while the assumptions underlying potentiodynamic polarization and Tafel extrapolation—such as activation-controlled kinetics and reasonably defined anodic and cathodic regions—are broadly applicable, deviations may arise due to temperature-induced surface instability. In cerium-containing systems, thermal effects can promote partial film breakdown or accelerated precipitation of cerium species, which may influence polarization responses and introduce uncertainty in the absolute corrosion current densities and Ecorr values. Therefore, the electrochemical parameters obtained at elevated temperatures are considered primarily as comparative indicators of corrosion tendency rather than strict kinetic constants. The electrochemical results of the SAC305 alloy were thoroughly evaluated in 3.5 wt% NaCl solution at various temperatures of 30, 40, and 50 °C with cerium inhibitor concentrations of 0, 200, and 700 ppm. Potentiodynamic polarization curves showed in Figs. 5, 6 and 7, corrosion rate trends in Fig. 8, and Nyquist plot (electrochemical impedance spectra) in Figs. 9, 10 and 11, also the corresponding parameters are showed in Tables 2, 3 and 4, show the combined effect of temperature and inhibitor concentration on the kinematics of corrosion and inhibition efficiency. The polarization curves were recorded over a sufficiently wide potential range to clearly identify the anodic and cathodic Tafel regions, ensuring reliable extrapolation of corrosion parameters.

**Table 5:** EIS measurements for SAC305 alloy in 3.5% NaCl media at different cerium concentrations and temperatures.

Inhibitor Conc.	Temperature ^0^C	Rs (Ω cm²)	*R*₂ = Rct (Ω cm²)	Y₀ = Qdl (Ω⁻¹ sⁿ cm⁻²)	*N*
0	30	20	2160	1.23 × 10⁻3	0.82
200	30	15	4765	6.66 × 10⁻⁴	0.86
700	30	10	8180	2.5 × 10⁻4	0.90
0	40	40	6460	1.2 × 10⁻³	0.82
200	40	35	7165	6.5 × 10^− 4^	0.86
700	40	25	575	2.5 × 10⁻^4^	0.90
0	50	40	1460	1.2 × 10⁻³	0.82
200	50	35	7765	6.5 × 10⁻⁴	0.86
700	50	25	775	2.5 × 10⁻⁴	0.90

### Potentiodynamic polarization response

The SAC305 solder alloy shows a rest potential of − 844.09 mV and a corrosion current density of 0.04 mA cm⁻² at 30 °C, as evidenced in Fig. 5; Table 2, corresponding to a corrosion rate of 20.83 m/y. When 200 ppm of cerium is added to 3.5% NaCl, the corrosion rate decreases markedly to 3.65 m/y and Icorr drops to 0.007 mA cm⁻², resulting in an inhibition efficiency (ηp) of 82.5%. Further improvement is observed at 700 ppm, where Icorr decreases to 0.004 mA cm⁻² and the corrosion rate to 2.08 m/y, yielding an inhibition efficiency of 90.0%. This significant enhancement suggests the formation of a compact and protective surface film at higher cerium concentration, effectively limiting chloride ion access to electrochemically active sites.

By extending the linear segments of the anodic and cathodic Tafel branches within ± 50 mV of Ecorr, where activation controlled kinetics dominate, corrosion current densities were determined. This approach is widely applied for corrosion rate evaluation and helps minimize errors associated with diffusion effects and surface film interference^22–24^. Deviations from linearity beyond this potential window are attributed to progressive surface film formation and partial passivation, particularly in cerium containing electrolytes. The fitting regions used for Tafel extrapolation are highlighted in the polarization plots to allow visual identification of the linear segments used for parameter extraction.

The strong dependence of Icorr on temperature and cerium concentration reflects genuine modifications in electrochemical behavior. At 200 ppm and 700 ppm Ce³⁺, the development of a Ce(OH)₃/CeO₂-based surface layer suppresses both anodic metal dissolution and cathodic oxygen reduction. At 30 °C, the simultaneous increase in βa and βc values confirms that the inhibitor affects both partial reactions, indicating a mixed-type inhibition mechanism consistent with previous studies on cerium-modified solder alloys in chloride media^25–31^.

The simultaneous reduction in anodic (βa) and cathodic (βc) Tafel slopes confirms that the inhibitor influences both partial reactions, indicating a mixed-type inhibition mechanism. This behaviour suggests that cerium-derived surface films restrict charge transfer at anodic dissolution sites as well as cathodic reaction zones, in agreement with previous observations for tin-based solder alloys in chloride media^30,31^.

At 40 °C (Fig. 6; Table 3), the corrosion rate of the uninhibited alloy increases to 22.83 m/y, with Icorr rising to 0.026 mA cm⁻², indicating accelerated electrochemical kinetics at elevated temperature. The addition of 200 ppm cerium reduces the corrosion rate to 10.43 m/y (ηp = 53.8%), while 700 ppm further lowers Icorr to 0.007 mA cm⁻² and provides an inhibition efficiency of 73.1%. Although inhibition remains significant, the protection efficiency is lower than at 30 °C, suggesting reduced film stability at higher temperature. The variation in βa and βc values continues to support a mixed-type inhibition behavior, with both anodic and cathodic processes being moderated by the inhibitor.

At 50 °C (Fig. 7; Table 4), the uninhibited SAC alloy exhibits a corrosion rate of 20.081 m/y with Icorr = 0.018 mA cm⁻². The addition of 200 ppm cerium shifts Ecorr slightly to − 534.09 mV and reduces Icorr to 0.011 mA cm⁻², corresponding to an inhibition efficiency of 40%. Increasing the concentration to 700 ppm decreases Icorr further to 0.007 mA cm⁻² and reduces the corrosion rate to 7.8 m/y, giving an efficiency of 60%. These results indicate that, although cerium continues to provide measurable protection at 50 °C, its effectiveness diminishes compared with lower temperatures, likely due to partial thermal destabilization of the protective cerium-based film^34,35^.

The moderate changes in anodic and cathodic Tafel slopes at 50 °C suggest reduced film compactness and enhanced dissolution kinetics, particularly for tin-rich phases. Similar temperature-related decreases in inhibitor performance have been reported for SAC and other lead-free solder alloys in chloride-containing environments^36,37^.

### Corrosion rate trends

It should be noted that the application of Tafel extrapolation assumes stable electrochemical conditions and well defined linear anodic and cathodic regions. In inhibitor containing systems, particularly at elevated temperatures, rapid surface film formation, partial breakdown, or dynamic adsorption–desorption processes can violate these assumptions. Under such circumstances, extrapolated corrosion current densities and derived inhibition efficiencies may exhibit nonphysical values, including negative efficiencies or exaggerated shifts in corrosion potential^38–41^. Therefore, the polarization parameters reported in this study are interpreted primarily to identify qualitative corrosion trends rather than to derive absolute kinetic constants.

Figure 8 summarizes the variation in corrosion rate as a function of inhibitor concentration and temperature. In the absence of inhibitor, the SAC305 alloy exhibits the highest corrosion rates at all temperatures, reflecting the unprotected surface. The addition of 200 ppm cerium significantly reduces the corrosion rate at each temperature, indicating effective protective film formation. At 700 ppm, the corrosion rate further decreases compared with 200 ppm, indicating improved protection at higher inhibitor concentration under the investigated conditions. Although corrosion rates increase with temperature, the overall concentration dependent trend remains consistent.

### Electrochemical impedance spectroscopy analysis

The impedance behaviour of the SAC305 alloy is illustrated by Nyquist plots in Figs. 9, 10 and 11. The impedance plots exhibit capacitive loops which are the characteristic of charge transfer-controlled corrosion procedures, connected with double-layer capacitance and charge transfer resistance. The loop diameter directly shows the variations in corrosion resistance, with larger diameters indicating lower corrosion rates^42–44^.

At 30 °C, the impedance loop diameter increases with the addition of 700 ppm of cerium inhibitor, whereas at 40 °C, the largest loop diameter is observed at 200 ppm of cerium. At 50 °C, higher impedance values are seen for the system without inhibitor addition and for the alloy containing 200 ppm cerium. These trends indicate that optimal inhibitor concentration depends on temperature and film stability.

The corrosion inhibition behavior is reflected in the charge transfer resistance (Rct) values obtained from the EIS measurements. An increase in Rct indicates improved corrosion resistance due to the formation of a protective inhibitor film on the metal surface. At 30 °C, the Rct value increases significantly with increasing cerium concentration, reaching a maximum at 700 ppm, indicating strong adsorption of inhibitor molecules and formation of a protective film^22,39,40^. However, at higher temperatures (40 and 50 °C), the Rct values decrease at 700 ppm, suggesting possible instability or partial breakdown of the protective film at excessive inhibitor concentration. The constant phase element (CPE) values decrease with increasing inhibitor concentration, indicating a reduction in double layer capacitance due to the adsorption of inhibitor molecules and the formation of a compact surface film^22,45–49^. These results suggest that moderate concentrations of cerium provide optimal corrosion protection, particularly at elevated temperatures.

### Inhibition mechanism and temperature effects

The inhibition efficiency clearly depends on both temperature and cerium concentration. At 30 °C, strong adsorption and the formation of a compact and stable surface film result in high inhibition efficiencies (up to 90% at 700 ppm). At 40 and 50 °C, although inhibition is still evident, the efficiencies decrease (maximum 73.1% at 40 °C and 60% at 50 °C), indicating reduced film stability and partial desorption at elevated temperatures. Thus, adsorption is more effective and protective at lower temperature, while increasing temperature promotes gradual weakening or structural modification of the cerium-based layer.

Cerium inhibits corrosion primarily through the formation of insoluble Ce(OH)₃/CeO₂ layers that preferentially precipitate at cathodic sites, thereby suppressing oxygen reduction reactions^25,27,28^. Hydroxide ions generated during cathodic oxygen reduction increase the local pH, promoting Ce(OH)₃ precipitation, which may subsequently oxidize to CeO₂. The resulting partially insulating barrier blocks active cathodic sites, reduces charge transfer, and indirectly decreases anodic metal dissolution. Because anodic and cathodic reaction rates are interdependent, suppression of the cathodic reaction leads to an overall reduction in corrosion rate, supporting cerium as a viable non-toxic alternative to chromate-based inhibitors^50^. The mixed changes observed in both βa and βc values across all temperatures further confirm that cerium acts as a mixed-type inhibitor.

However, corrosion protection is strongly concentration and temperature dependent. At 30 °C, increasing Ce³⁺ concentration from 200 ppm to 700 ppm enhances protection, indicating improved film compactness and surface coverage. At 40 and 50 °C, although 700 ppm still provides better protection than 200 ppm, the overall efficiency decreases compared with 30 °C, suggesting that elevated temperature limits film stability. Under such conditions, faster precipitation kinetics and possible film porosity may reduce adhesion and uniformity. In some cases, cerium species may partially precipitate in the bulk electrolyte rather than forming a continuous surface layer, which can contribute to localized attack^25,26,29,51^. Similar temperature- and concentration-dependent behavior has been reported for SAC alloys in 3.5 wt% NaCl solution, where inhibition efficiency declines at higher temperatures due to film heterogeneity and susceptibility of Sn- and Cu-rich phases to localized corrosion^28^.

The fluctuations observed in electrochemical parameters can also be attributed to the dynamic nature of the native SnO₂/SnO surface film on the SAC alloy, whose thickness and compactness are influenced by both temperature and the presence of cerium ions, as reported for Sn-based solder systems^52–55^. Temperature-induced modifications of this oxide layer may further affect charge-transfer resistance and polarization characteristics.

#### Surface morphology and elemental analysis

**Fig. 12:**
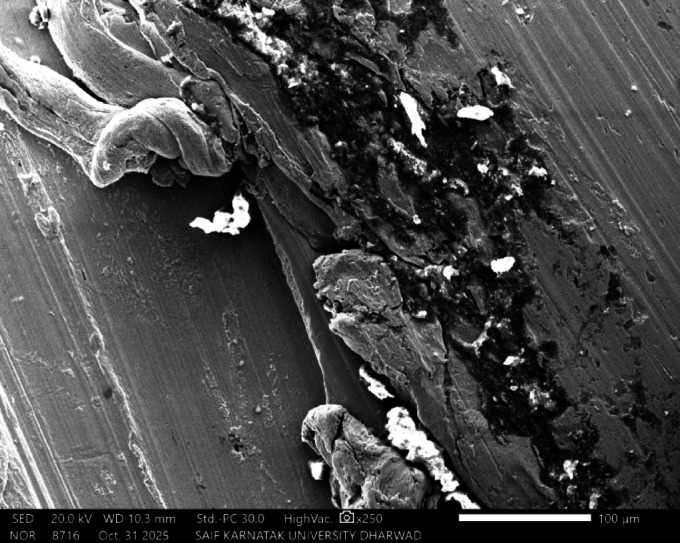
SEM image of SAC305 alloy in 3.5% NaCl solution.

**Fig. 13:**
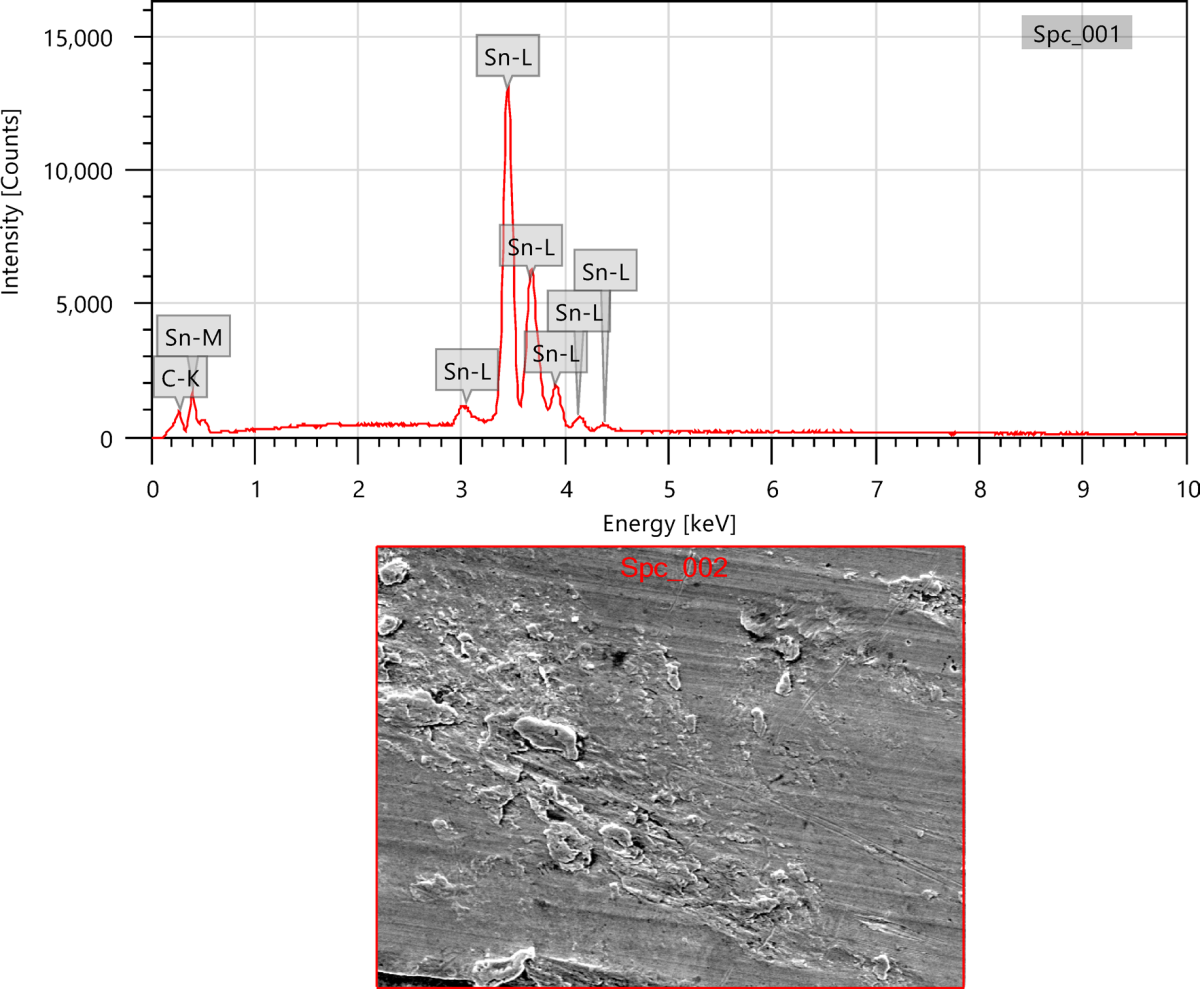
SEM and EDX image of SAC305 alloy in 3.5% NaCl solution.

**Fig. 14:**
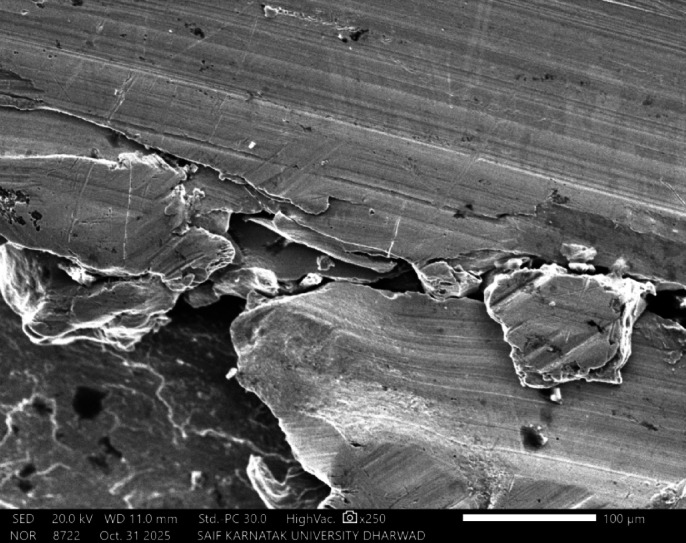
SEM image of SAC305 alloy in 3.5% NaCl solution with 200 ppm of Cerium.

**Fig. 15:**
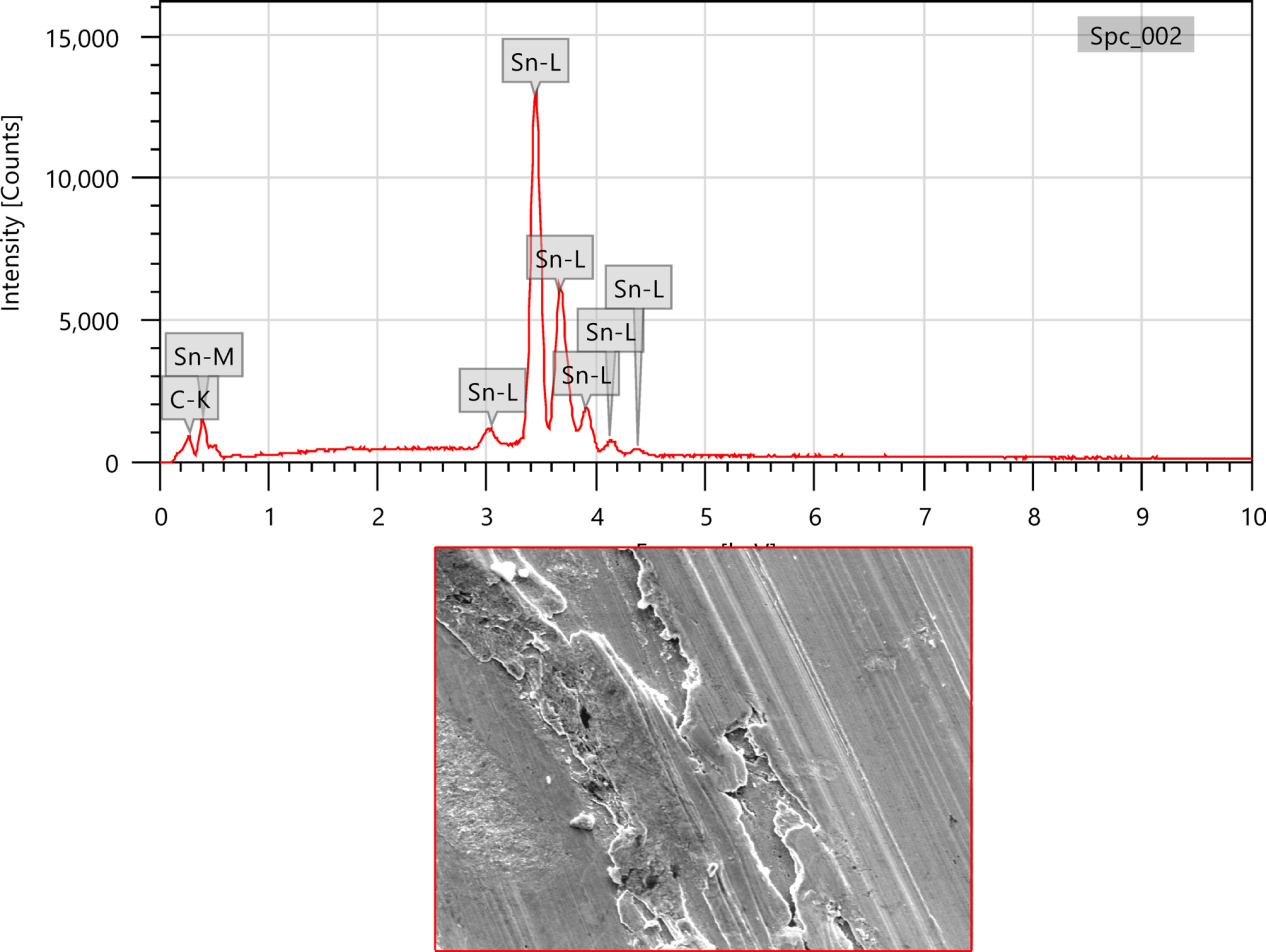
SEM and EDX image of SAC305 alloy in 3.5% NaCl solution with 200 ppm of inhibitor.

**Fig. 16:**
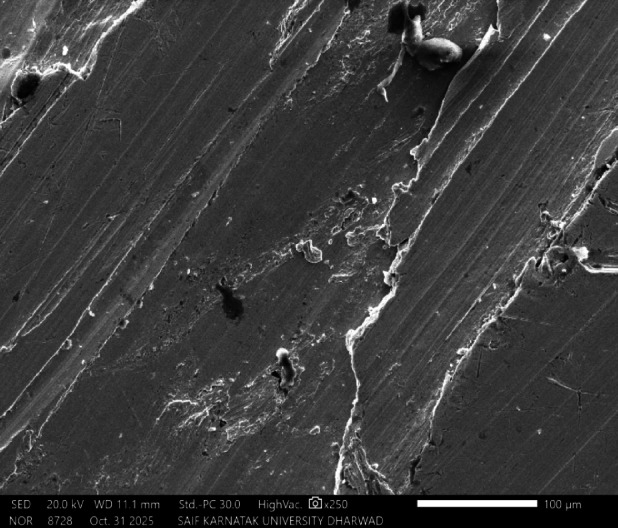
SEM image of SAC305 alloy in 3.5% NaCl solution with 700 ppm of Cerium.

**Fig. 17:**
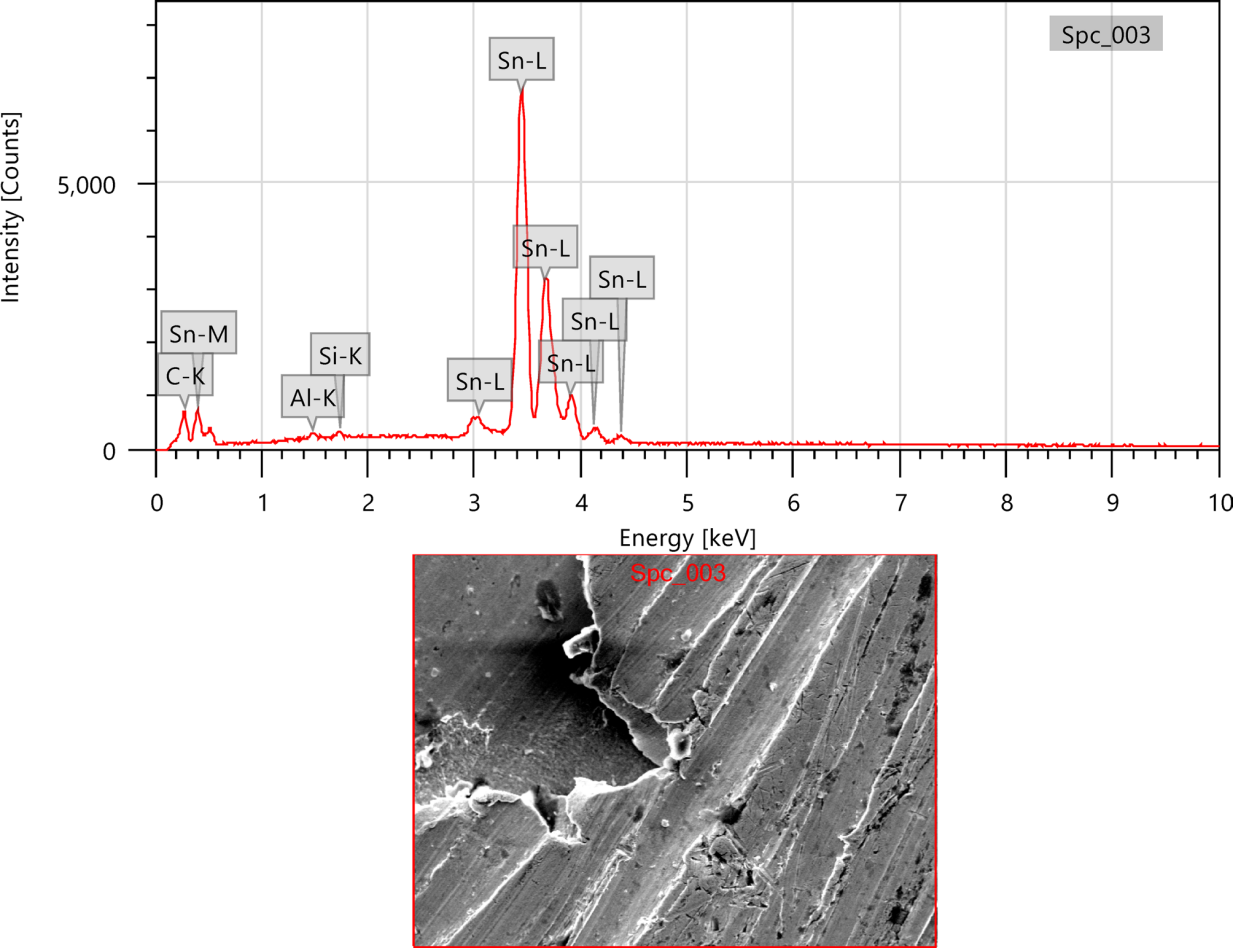
SEM and EDX image of SAC305 alloy in 3.5% NaCl solution with 700 ppm of Cerium.

SEM micrographs of the SAC305 alloy surface after corrosion exposure reveal noticeable changes in surface morphology compared to the uninhibited condition. The presence of inhibitor is associated with a relatively smoother surface and reduced severity of localized corrosion features, suggesting inhibitor induced surface modification (Figs. 12, 13, 14, 15, 16, 17).

EDS analysis of the corroded surfaces primarily detects Sn, Cu, and O, consistent with the formation of tin and copper based oxides during corrosion exposure. Cerium related peaks are not clearly observed in the EDS spectra, indicating that any cerium containing species if present exist in very low concentrations, as ultrathin surface deposits, or in a highly dispersed form below the detection limit of the technique^56–58^.

Accordingly, SEM/EDS results are interpreted qualitatively to indicate possible surface modification and altered oxide characteristics in the presence of the inhibitor, rather than the confirmed formation of a continuous CeO₂/SnO₂ protective film.

#### XRD analysis

**Fig. 18:**
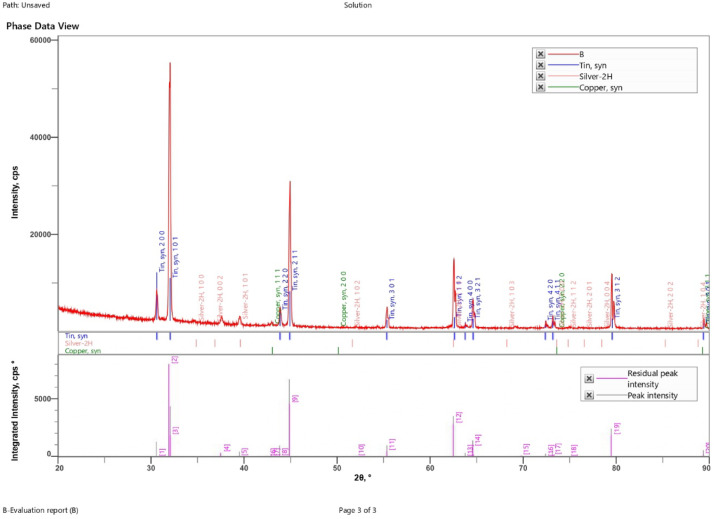
XRD of SAC305 alloy in 3.5% NaCl solution.

**Fig. 19:**
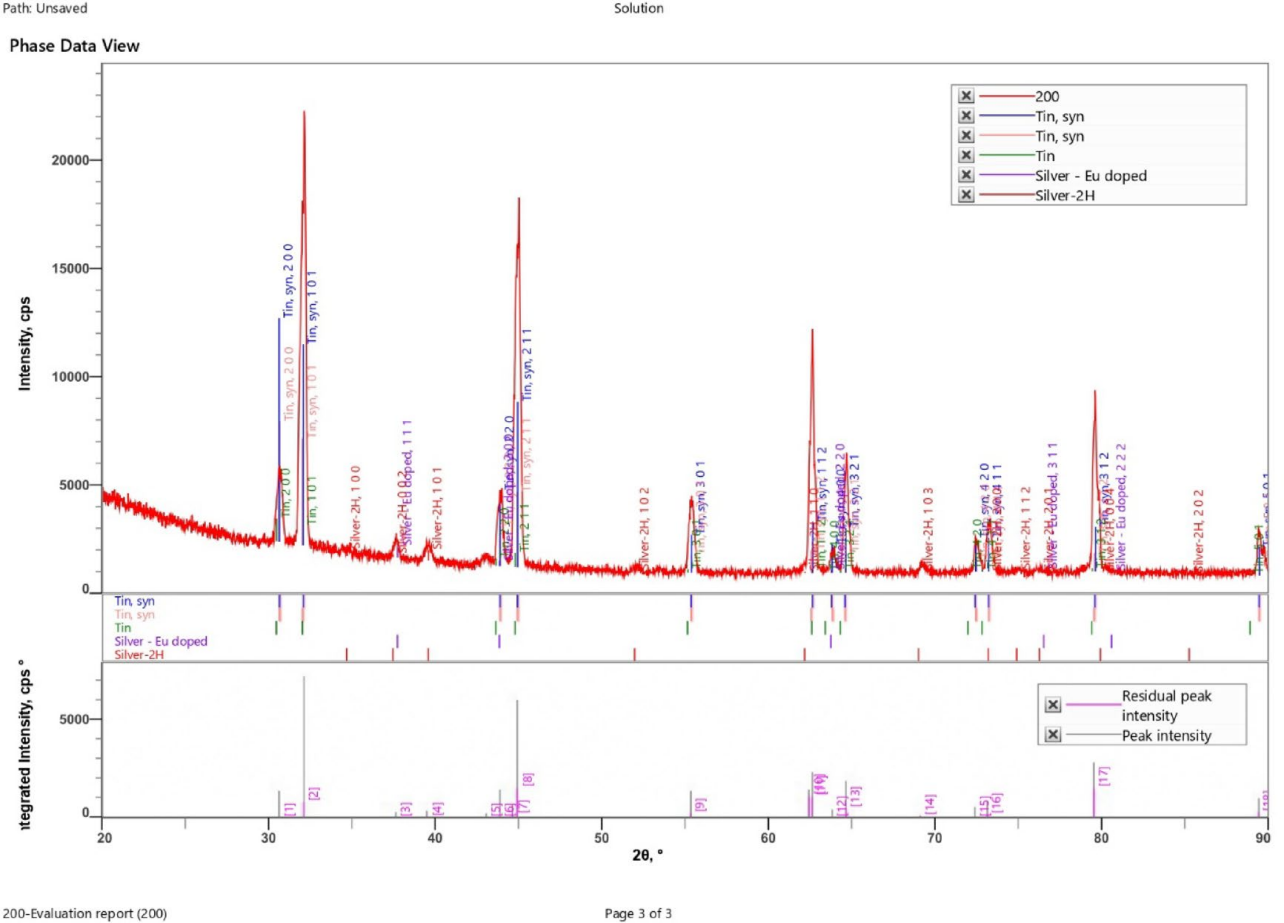
XRD of SAC305 alloy in 3.5% NaCl solution with 200 ppm of cerium.

**Fig. 20:**
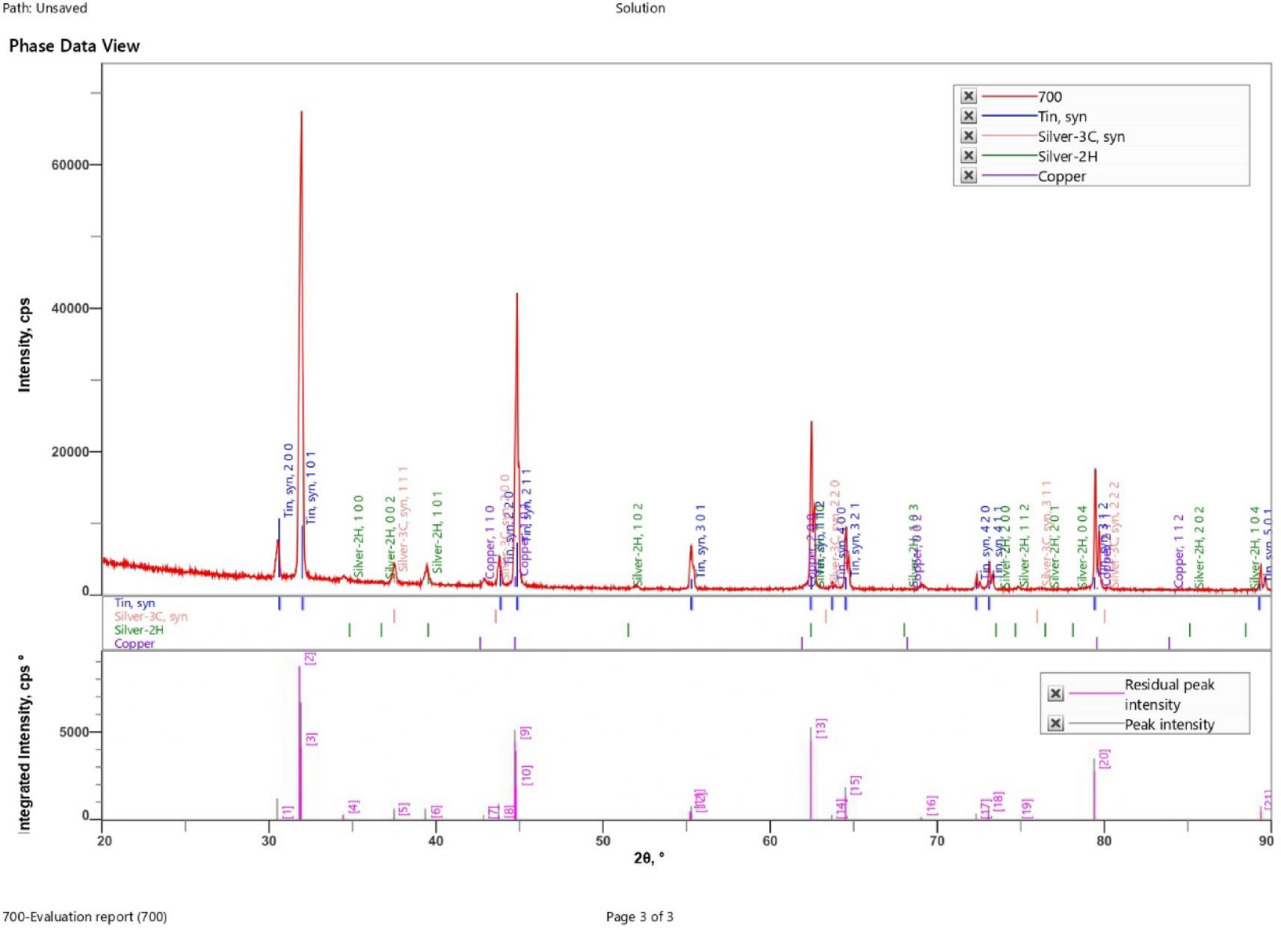
XRD of SAC305 alloy in 3.5% NaCl solution with 700 ppm of cerium.

X-ray diffraction (XRD) patterns of the as-prepared and corroded SAC305 alloy samples confirm the retention of the characteristic Sn–Ag–Cu phases after exposure to the corrosive environment, irrespective of inhibitor concentration. The dominant diffraction peaks corresponding to β-Sn and intermetallic phases remain unchanged, indicating that the fundamental bulk phase constitution of the alloy is preserved.

Minor reflections attributable to surface oxides such as SnO₂ and Cu₂O are weak and limited in intensity, suggesting that oxidation remains confined to near surface regions^58,59^.Given the surface-localized nature of corrosion and the absence of quantitative line-broadening or phase refinement analysis, XRD results are interpreted qualitatively for phase identification and confirmation of bulk phase stability only. No conclusions regarding lattice strain, crystallite size, or crystallinity evolution are drawn from the present data^60,61^.

The absence of additional diffraction peaks following corrosion exposure indicates that no new bulk phases are formed and that corrosion processes do not significantly alter the underlying crystalline structure of the SAC305 alloy^62^.

Accordingly, XRD is employed in this study as a qualitative tool for phase identification and bulk phase stability assessment, rather than for quantitative microstructural analysis.

X-ray diffraction (XRD) patterns obtained from the as-prepared and corroded SAC305 alloy samples (Figs. 18, 19 and 20) confirm the preservation of the alloy’s crystalline phases. Strong diffraction peaks corresponding to Sn, Ag, and Cu are observed in all samples, indicating that the fundamental phase constitution of the alloy remains unchanged following corrosion exposure. Minor reflections attributed to SnO₂ and Cu₂O suggest surface oxidation, which contributes to the formation of thin oxide layers that assist in corrosion inhibition^58,59^.

Overall, the XRD results demonstrate that the Sn–Ag–Cu alloy maintains phase purity while developing stable oxide layers during corrosion exposure. These characteristics, combined with the observed microstructural stability, support the improved corrosion resistance and structural integrity of the alloy under the investigated conditions.

## Conclusion

700 ppm cerium-based inhibitor significantly improved the corrosion resistance of the SAC305 (Tin-Silver-Copper) solder alloy in a 3.5% NaCl solution. This performance was notably effective at 30 °C and provided respectable protection even at higher temperatures up to 50 °C. The enhanced performance is attributed to cerium ions’ capacity to adsorb on the alloy surface and promote the growth of a stable oxide/hydroxide coating, which serves as an impenetrable barrier against aggressive chloride attack. This suggests cerium acts as a stabilizing agent, significantly prolonging the service life of the passive film on SAC305 in harsh conditions.

Practically, these results are very relevant to electronic packaging applications, where SAC305 is rapidly replacing banned lead-based solders. In chloride-rich environments (e.g., coastal regions, humid environments, or marine electronics), this cerium treatment directly addresses the primary failure mode of SAC305: galvanic corrosion and pitting caused by salt. By stabilizing the passive film and blocking cathodic sites, the cerium inhibitor provides a sustainable and eco-friendly method to ensure long-term solder joint reliability and structural integrity without compromising the material’s critical electrical or mechanical properties. The demonstrated efficacy at 700 ppm also underscores the economic viability and ease of industrial adoption.

## Data Availability

Corresponding author agrees to provide the data upon reasonable request.
